# A Case of the Foramen Ovale Found Not Closed on the Dissection of a Soldier, with Its Previous History, and Observations; as Reported to the Director General of the Army Medical Department, in December Last

**Published:** 1828-10

**Authors:** Andrew Blake

**Affiliations:** Member of the Royal College of Surgeons, and Surgeon to the 7th Dragoon Guards.


THE LONDON
### Medical and Physical Journal.

N° 356, VOL. LX.] OCTOBER, 1828. [N° 28, *New Series.*

For many fortunate discoveries in medicine, and for the detection of numerous errors, the world is indebted to the rapid circulation of Monthly Journals; and there never existed any work, to which the Faculty, in Europe and America, were under deeper obligations, than to the *Medical and Physical Journal of London*, now forming a long, but an invaluable, series.—RUSH.

### ORIGINAL PAPERS,

AND

### CASES OBTAINED FROM PUBLIC INSTITUTIONS AND OTHER AUTHENTIC SOURCES.

### FORAMEN OVALE.

*A Case of the Foramen Ovale found not closed on the Dissection of a Soldier, with its previous History, and Observations; as reported to the Director General of the Army Medical Department, in December last.* By ANDREW BLAKE, M.D. Member of the Royal College of Surgeons, and Surgeon to the 7th Dragoon Guards.

A DRAGOON, aged twenty-one years, of the sanguineous temperament, a robust make, and not brought up to any trade, at the expiration of two years and ten months' service, reported himself sick, on the 8th of September, 1826. On examination, he appeared to labour under slight febrile symptoms, and stated that for some time past he had been subject to what he called weaknesses, but which appeared to have been attacks of syncope cardiaca, during which he became insensible, and the face, but particularly the lips, assumed a dark colour. They were not, however, of long duration, and their effects were quickly dissipated.

Soon after his admission, the febrile symptoms increased, and he seemed to labour under an inflammatory affection of the viscera of the left side of the thorax, in which the heart or pericardium participated: at least, he suffered from the principal diagnostic symptoms of these complaints, namely, pain in the cardiac region, attended with a rapid irregular pulse, cough, and a sense of extreme uneasiness and suffocation, which were all greatly augmented by the horizontal position. He attributed his state to the effects of a fall he met with while exercising his horse, soon after he began to learn to ride; for which it appears my predecessor, Dr. Rose, found it necessary to bleed and blister him. During the whole of his life, previous to his having fallen from the horse, he enjoyed uninterrupted health.

For these alarming symptoms I bled him twice copiously, administered digitalis, with calomel and opium, and kept a blister open on the chest. These remedies arrested the progress of the disease, but palpitation of the heart and irregularity of the pulse continued, attended with occasional attacks of syncope cardiaca.

The system was now brought slightly under the influence of mercury. He was put on milk diet, and digitalis was given in large doses. Under this treatment he seemed to recover gradually, and was discharged to duty on the 11th of October following, in apparent good health.

He continued well, and able to perform all the duties of a dragoon without inconvenience, up to the month of April, 1827, when he embarked at Dublin for Liverpool, on his way to Coventry. On my arrival there soon after, I found him in hospital, suffering from cough, with muco-purulent expectoration. The lips were of a livid hue; he complained of confusion in his ideas, and appeared listless and inactive. These symptoms gradually increased, and were latterly accompanied with hectic exacerbations and occasional diarrhœa, which alternated with colliquative perspirations.

On the 27th May, 1827, I reported his case to the director general as one of phthisis complicated with disease of the heart, with a view to obtain his sanction to the patient's return to his native place, which was immediately granted; but his situation unexpectedly became such as to preclude the possibility of indulging him in the object he so much wished to obtain. From being able to walk into the country, which he was in the habit of doing daily, he became suddenly so weak as not to be able to get out of bed; and, without suffering from any other apparent symptom than those already mentioned, attended with extreme debility, he expired on the fourth day of his confinement to bed, without even being previously affected with the mucous wheeze.

In my report to Sir James M'Grigor, above alluded to, I stated the result of having explored the thorax to have been as follows: " Percussion elicits a dull sound from the whole of the lower part of the left side of the thorax. The stethoscope conveyed the respiratory murmur very indistinctly from the same parts. On exploring the cardiac region particularly, the rythm was observed to be occasionally irregular; the impulse on the left side was more considerable, while the sound was nearly equal on both sides, though greater than natural. Pulse generally very quick, and sometimes irregular."

The body was examined twenty hours after death by Dr. Stewart, now physician to the forces, and myself: it presented an emaciated appearance, and a matter of a bilious and fetid nature flowed from the mouth, while the abdomen was distended as if by flatus.

The abdominal cavity was first opened, from which there issued a considerable quantity of sero-purulent matter. The surface of the intestines generally presented the appearance of having been inflamed, and were covered with thick and adhesive purulent fluid ; large quantities of which, from gravitation, had accumulated in the lumbar regions. The marks of inflammation were not vivid, but of a very pale rose colour. The intestines presented nothing remarkable in their structure; but the peritoneum covering the right lobe of the liver, and lining the contiguous parts, seemed also to have undergone inflammation, and was thickly covered with the same kind of viscid pus-like fluid as already spoken of. A superficial stratum of the parenchyma of this lobe appeared to have participated in the inflammatory action, but the remainder of its substance was perfectly healthy. It was rather remarkable that this inflammation terminated abruptly at the falciform ligament, and did not extend to the left lobe or its covering. No other morbid appearances were discovered in this cavity.

On removing the sternum, universal adhesions were brought into view on both sides of the thorax, but on the left they were of a much more dense and firm structure than on the right. In the cavity of the former the contents appeared as one mass, from being so intimately blended together by the effects of adhesive inflammation, more especially on the anterior surface of the pericardium, where large quantities of coagulable lymph had been extravasated. The structure of the lung on this side, when cut into, shewed extensive devastation from the ravages of tubercles and vomicæ ; while that on the right side, although slightly tuberculated, appeared still to be capable of performing the respiratory function. Having laid open the pericardium, an unusual quantity of serum was found in its cavity, and the right sinus of the heart appeared enlarged and dilated. On removing the heart, together with as much of the great vessels as were thought necessary, its minute examination was commenced by laying open the right sinus from the inferior cava to the extreme point of its auricular portion, by which the right side of the septum between the auricles was exposed, as also the Eustachian valve, which in this subject was remarkably beautiful. On examining the site of the fossa ovalis, a perfect and well-defined opening was observed at its upper and posterior part, of size sufficient to admit the largest goosequill, and leading in an oblique direction through the coats of the inter-auricular partition from right to left, forming a canal nearly a quarter of an inch in length, and thus preserving by its obliquity somewhat of a valvular character. At this stage of the dissection, the valve of the coronary vein was also observed: it was larger than usual, and beautifully delicate in its structure. There was now likewise an opportunity of observing that, independently of the enlargement of this auricle, its structure was extremely thin and relaxed. The right ventricle exhibited no remarkable appearance; the left auricle was also natural, save the opening already spoken of; but the left ventricle, in its external appearance and internal capacity, was corrugated and contracted, while its walls were strong, firm, and hard to the feel, evincing a comparative state of hypertrophy with regard to the other parts of this organ. No further effects of disease were found in the prosecution of this post-mortem investigation.

A preparation was made of the heart, displaying its peculiarities of structure: it accompanied this report, and is deposited in the army medical museum at Chatham, by order of the director general.

*Observations.*—It is difficult to account for the morbid appearances which the abdominal cavity presented in this case, as they were produced without any premonitory symptoms having manifested themselves, save occasional diarrhœa, with which the patient was not troubled for some days previous to his death. They appeared to me to have been the effect of a last effort of nature to establish a metastasis in favor of the more important organs already engaged, and which, being too great for the constitution to support in its reduced state, was the immediate cause of the sudden and fatal termination.

With respect to the opinions and prognosis which the symptoms of this case were likely to suggest, more particularly with regard to the state of the heart, I think it will be allowed they must have been very uncertain; and I am of opinion that whenever a similar case occurs,—that is to say, when a like malformation of the adult heart is combined with acute inflammation of the lungs on the left side,—although the stethoscope may enable us to discover the disorder in the organs of respiration, yet the heart or pericardium will always be supposed to be materially implicated in the inflammatory action which is going on.

In my yearly report to the director general, dated December 1826, at the conclusion of my observations on this man's first attack of illness, I expressed myself thus: "I must confess that I felt a good deal alarmed for the safety of my patient, as I feared, from the long-continued irregularity of the pulse, that structural disease must have existed: however, I observed at times that the pulse resumed its regularity; which circumstance assured me that the integrity of the heart and its appendages had been preserved, and that these symptoms only arose from some functional derangement of that important organ. The cases quoted in Good's work and in the Parisian hospital reports, tended much to give me confidence in my prognosis."

From what has been stated it will appear evident that, although I was correct as to my prognosis in this case, as far as regarded the integrity of the heart from the effects of active inflammation, I was not aware of the structural malformation which existed in the communication through the auricular septum, the oblique direction of which, acting as a valve, prevented, under ordinary circumstances, the development of any symptom which was capable of denoting its existence. I therefore hold this case to be of particular interest, inasmuch as it will serve to render practitioners cautious in forming a prognosis, lest such a combination of circumstances as are here alluded to may take place, and prevent them from pronouncing too hastily (because the irregularity of the pulse may be only occasional,) that their patient does not labour under some structural malformation of the heart.

Had the subject of this report lived a monastic life, where little or no bodily exertion would be required, he might have obtained old age without the irregularity being observed; but the fall he met with from his horse, and the necessary exertion and gêne which learning to ride in a cavalry regiment requires, appear to have forced the blood in an improper course, and excited the symptoms of deranged circulation already described. All such irregularities, under particular circumstances, must necessarily produce concomitant congestion, and thus gradually excite other structural changes in the organ in question, as well as inflammation in the lungs, more particularly where there is a tubercular disposition, as appears to have existed in this case. Indeed, whenever such a communication exists, which, owing to its valvular formation, may perhaps not serve as such until the patient shall have made some violent exertion, and is attacked with acute inflammation of the lungs, (possibly on the left side,) I am not acquainted with any diagnostic mark which, during the inflammatory action, will prove that the heart or its appendages are not equally engaged with the lungs. It is fortunate, however, that, in a practical point of view, the diagnosis is unimportant, as the treatment required in either case must be similar.

The preparation which accompanied this paper appeared also interesting in exemplifying, by the disposition to valvular formation which it exhibited, parts that in ordinary subjects are not so well marked; and, notwithstanding the following observation of that able anatomist, the late Mr. SHAW, viz. "this is so common that we cannot attach much importance to it," I took the liberty of offering it to Sir James M'Grigor for the interesting museum formed under his auspices at Chatham. Mr. Shaw also says he discovered in the septum auriculorum of the heart of a strong drayman, who died from rupture of the aorta, and who did not appear to have suffered from any affection that could be referrible to the state of the heart, an opening which would admit four fingers, although no valvular contrivance was observed in this case. This, in my opinion, might well be placed in the collection of "*cas rares*;" and the non-existence of symptoms denoting deranged circulation during lifetime can only be accounted for by supposing the equilibrium of size and power between the auricles to have been most accurately preserved, and consequently that the contracting power at one side of the heart did not prevail over that of the other.

Neither MORGAGNI nor Dr. BAILLIE give any specific example in their writings of the peculiarity in question having been found in an adult; and MASON GOOD, in his work, says, in the article on *Cyania*, "We may confidently assert that, whenever so large a portion of venous blood is thrown into the arterial circulation as to give a blue tinge to the lips, or the skin generally, all the functions will be performed feebly, and there is great danger that the infant will never reach the age of puberty."

It is to be remarked, in the case which forms the subject of this paper, that, previous to the fall alluded to, the individual was apparently healthy: consequently, it is to be supposed that the structural disease manifested by the dilatation of the right auricle, and hypertrophy of the left ventricle, must have been of subsequent formation; as, otherwise, it is more than probable irregularity of the pulse, and more or less cyania, would have been the consequence at an earlier period, owing to the disproportion which would have existed between the chambers of the heart.

*Manchester Barracks;*

*August 5th,* 1828.

